# 47629 Contribution of Auditory Function to Falls Risk in Adults with Vestibulopathy

**DOI:** 10.1017/cts.2021.755

**Published:** 2021-03-30

**Authors:** Ryan J. Huang, Carl F. Pieper, Heather E. Whitson, Douglas B. Garrison, Kristal M. Riska

**Affiliations:** 1 Duke University School of Medicine; 2 Duke University School of Medicine, Division of Biostatistics and Bioinformatics; 3 Duke Center for the Study of Aging and Human Development; 4 Duke University School of Medicine, Department of Head and Neck Surgery & Communication Sciences

## Abstract

ABSTRACT IMPACT: Findings from this study will better characterize the role of hearing loss in falls risk among patients with vestibulopathy and identify groups that are most at risk for falls. OBJECTIVES/GOALS: Vestibular dysfunction and hearing loss are independent risk factors for experiencing falls. The purpose of this study is to determine the extent, if any, to which hearing loss contributes to falls in patients with concomitant vestibular dysfunction presenting to a specialty vestibular clinic. METHODS/STUDY POPULATION: A retrospective chart review of patients ≥18 years who underwent vestibular evaluation at our institution from June 1, 2015 to October 7, 2020 will be conducted. Patients who underwent vestibular evaluation also received audiologic evaluation and degree of hearing loss will be characterized by the 4-frequency pure-tone average (0.5, 1, 2, and 4 kHz) of the better hearing ear. Falls status will be determined by the response to the following question administered at clinic-check in, ‘Have you fallen in the last 90 days?’ Demographics, comorbidities, and falls-associated medications will also be collected. RESULTS/ANTICIPATED RESULTS: A total of 3,265 unique patients who underwent vestibular evaluation in the study time period were identified. Patients will be categorized into discrete groups (benign paroxysmal positional vertigo, unilateral hypofunction, bilateral hypofunction, central dysfunction, and normal) based on laboratory results. Regression models will be developed to evaluate the potential association between degree of hearing loss and falls in patients with different types of vestibular dysfunction, while adjusting for demographics, comorbidities, and falls-associated medications. DISCUSSION/SIGNIFICANCE OF FINDINGS: Findings from this study will better characterize the role of hearing loss in falls risk among patients with vestibulopathy and identify groups that are most at risk for falls. This study may potentially indicate the importance of hearing evaluation in the work-up of patients with vestibulopathy.

